# Molecular evidence for increased regulatory conservation during metamorphosis, and against deleterious cascading effects of hybrid breakdown in *Drosophila*

**DOI:** 10.1186/1741-7007-8-26

**Published:** 2010-03-31

**Authors:** Carlo G Artieri, Rama S Singh

**Affiliations:** 1 Department of Biology, McMaster University, Hamilton, Ontario, L8S4K1, Canada; 2 Laboratory of Cellular and Developmental Biology, National Institute of Diabetes and Digestive and Kidney Diseases, National Institutes of Health, Department of Health and Human Services, Bethesda, Maryland, 20892, USA

## Abstract

**Background:**

Speculation regarding the importance of changes in gene regulation in determining major phylogenetic patterns continues to accrue, despite a lack of broad-scale comparative studies examining how patterns of gene expression vary during development. Comparative transcriptional profiling of adult interspecific hybrids and their parental species has uncovered widespread divergence of the mechanisms controlling gene regulation, revealing incompatibilities that are masked in comparisons between the pure species. However, this has prompted the suggestion that misexpression in adult hybrids results from the downstream cascading effects of a subset of genes improperly regulated in early development.

**Results:**

We sought to determine how gene expression diverges over development, as well as test the cascade hypothesis, by profiling expression in males of *Drosophila melanogaster*, *D. sechellia*, and *D. simulans*, as well as the *D. simulans *(♀) × *D. sechellia *(♂) male F1 hybrids, at four different developmental time points (3rd instar larval, early pupal, late pupal, and newly-emerged adult). Contrary to the cascade model of misexpression, we find that there is considerable stage-specific autonomy of regulatory breakdown in hybrids, with the larval and adult stages showing significantly more hybrid misexpression as compared to the pupal stage. However, comparisons between pure species indicate that genes expressed during earlier stages of development tend to be more conserved in terms of their level of expression than those expressed during later stages, suggesting that while Von Baer's famous law applies at both the level of nucleotide sequence and expression, it may not apply necessarily to the underlying overall regulatory network, which appears to diverge over the course of ontogeny and which can only be ascertained by combining divergent genomes in species hybrids.

**Conclusion:**

Our results suggest that complex integration of regulatory circuits during morphogenesis may lead to it being more refractory to divergence of underlying gene regulatory mechanisms - more than that suggested by the conservation of gene expression levels between species during earlier stages. This provides support for a 'developmental hourglass' model of divergence of gene expression in *Drosophila *resulting in a highly conserved pupal stage.

## Background

Studies in the field of evolutionary developmental biology have highlighted an important role for the divergence in patterns of gene regulation in shaping species-specific developmental outcomes. However, they have generally focused on a few loci, with the intent of mapping the precise changes in *cis *regulatory sites that are responsible for altered phenotypes [1,2]. Conversely, large-scale interspecific comparative gene regulation studies in the context of development are lacking, despite a growing body of speculation about the importance of divergence in regulatory networks in determining broad evolutionary patterns [3,4]. Such comparative studies are crucial to a more complete synthesis of evolution and development, as theoretical models of evolutionary processes are ultimately derived from the attempt to explain general patterns, rather than from case studies [5]. A number of researchers have used interspecific hybrids in order to study patterns of gene expression divergence at the level of the transcriptome, typically with the intent of elucidating the incompatible divergence of regulatory factors responsible for post-zygotic reproductive isolation leading to speciation (reviewed in [6]). Such hybrids offer us the chance to reveal incompatible divergence between regulatory elements that are masked by stabilizing selection acting to maintain similar expression levels in the parental species [7]. These studies have found a wide quantitative divergence of gene expression levels between hybrids and (same sex) members of their parental species, manifested as an improper expression of genes within hybrids relative to both parental species. The majority of these misexpressed genes are underexpressed relative to the parents, which is thought to result from a loss-of-function phenotype in hybrids caused by the incompatible divergence of gene regulatory elements, either in *cis *or *trans*. A potential caveat to expression studies employing interspecific hybrids is that they have generally examined only a single developmental stage, typically the adult. Hybrids are often characterized by the breakdown of various developmental systems, such as atrophied or absent germlines or heterosis of particular tissues/organs [8,9]. Such observations have led to the suggestion that widespread misexpression of genes in adults may not reflect the equally widespread incompatible divergence of regulatory elements during this stage. Rather, they may result from incompatibilities occurring among a smaller number of genes upstream in ontogenic hierarchies that have complex cascading effects that manifest themselves as an increasing proportion of misexpressed genes as ontogeny progresses [6]. This increase in the proportion of misexpressed genes may result from two non-mutually exclusive mechanisms: (1) improper regulation of genes occurring early in development may lead to improper development of particular tissues creating allometric differences in mRNA abundance relative to the parental species. (2) Improper regulation of early genes may lead to improper regulation of their downstream targets, which propagates throughout the developmental regulatory network. Irrespective its ultimate cause, we shall refer to this hypothesis as the 'cascade model' of hybrid misexpression.

The notion that changes occurring early in development are likely to lead to deleterious cascading effects in later stages is the most popular explanation for the observation that species are generally more similar to one another morphologically in earlier developmental stages compared to later developmental stages (known as Von Baer's [10] 'third law of development' [11,12]). Generally termed 'developmental constraint' [12], this hypothesis invokes the notion that purifying selection will be stronger when acting on early-expressed, developmentally integrated genes [13]. Evidence for the developmental constraint hypothesis has come from the observation that genes expressed early in development tend to be more conserved at the sequence level than those expressed later [14-16]. More recently, it has been suggested that, while constraint may explain conservation of sequence and structure in early development, a greater opportunity for selection in later stages, engendered by such features as greater organismal mobility, complexity of behavior and sexual reproduction, may also contribute to the greater level of divergence seen among species' adults [16] - a theory originally proposed by Darwin [17]. It should also be noted that, while Von Baer's third law holds generally, it has been established that interspecific divergence does not increase monotonically over the entire course of development; rather, the earliest stages of ontogeny in vertebrates, for example, can vary substantially between species [12]. This has led to the proposal of a 'developmental hourglass' model of ontogenic divergence, wherein certain stages of development are more highly conserved as a result of a greater integration of complex regulatory interactions compared to those occurring either earlier or later - typically those stages during which organogenesis begins [12,18] (see also [19] for criticism of this model).

In order to test the predictions of the cascade model of hybrid misexpression, as well as to address whether Von Baer's law holds at the level of gene expression, we conducted *Drosophila melanogaster *cDNA microarray-based expression profiling of males of *D. melanogaster*, *D. sechellia *and *D. simulans*, at four synchronized developmental time points [3rd instar larval (larval), early pupal, late pupal and newly-emerged adult (adult)]. *D. simulans *and *D. sechellia *shared a most recent common ancestor (MRCA) ~0.5 - 1.0 million years ago (MYA) and form a clade that shared an MRCA with *D. melanogaster *approximately 5.4 MYA [20,21]. In addition, we also performed the same analysis on the male interspecific F1 hybrids of the *D. simulans *(♀) × *D. sechellia *(♂) cross (hereafter simply called 'hybrids'). We sought to test two specific hypotheses:

1. Does interspecific divergence in gene expression level increase over development (as has been observed for coding sequence divergence) or is expression more conserved during a particular developmental stage, indicating greater regulatory integration during a particular part of the life cycle?

2. Is such divergence also manifested in interspecific hybrids via an increasing proportion of misexpressed genes over subsequent stages of development, directly revealing regulatory divergence?

We find evidence that gene expression levels, when compared between pure species, follow a pattern of greater conservation in the earlier stages, as has been previously observed at the level of gene sequence and morphology. However, our results in hybrids do not support the developmental cascade model of misexpression: rather, we find ontogenic stage-specific breakdown of expression with the fewest misexpressed genes observed during the late pupal time point. Our data provide important insights for research exploring both the evolution of gene regulation in the context of development and the genetics of speciation, as it appears that phenotypic patterns of divergence observed in interspecific comparisons may mask more complex divergence occurring at the molecular level [22].

## Results

### Within-species expression patterns over ontogeny

We first sought to compare how patterns of gene expression varied within species over the course of our four sampled developmental time points. Restricting our comparison to the 2,006 genes that were detectibly expressed at all four time points in the three species and the hybrids, we found that 64.2% (1,287), 82.2% (1,649), 62.2% (1,247) and 57.9% (1,162) of genes varied significantly in the expression level over the course of the sampled developmental interval in *D. melanogaster*, *D. sechellia*, *D. simulans *and the hybrids, respectively (Additional File 1 contains supplementary methods and analysis, and tables containing raw data and the results of statistical analyses are found in Additional File 2). The proportion of genes that varied significantly during development in *D. sechellia *was significantly greater than that of the other two species and the hybrids (χ^2 ^test, 1 degree of freedom [df], *P *= 3.709214 × 10^-6^, 1.179722 × 10^-7^, and 1.642797 × 10^-11 ^for the comparison with *D. melanogaster*, *D. simulans*, and the hybrids, respectively; Bonferroni correction was applied to all pairwise tests). However, no other pairwise comparisons were statistically significant (*P *> 0.05). The relationships among genes varying among the three pure species or the parental species and the hybrids are shown in Venn diagram form in Additional File 3. Numerous lines of evidence suggest that *D. sechellia *arose from a relatively recent island colonization event, perhaps having gone through a severe bottleneck, and have since maintained low effective population sizes [20,23], leading to a reduced level of intraspecific polymorphism relative to the other two pure species. A reduced level of intraspecific polymorphism in expression level could lead to reduced estimates of between-replicate variability on the microarrays, thus improving our statistical power to detect significant differences. An inspection of the distributions of between replicate variances in our array data revealed that, indeed, *D. sechellia *showed significantly reduced between replicate variance compared to the other two species and the hybrids during the three earliest sampled times in our ontogenic interval (Kruskal-Wallis rank sum test, *P *< 2.2 × 10^-16^; Additional File 4). Therefore, we generated a set of random array values for these time points, retaining the means of *D. sechellia *while scaling their variance to that estimated from *D. simulans *(see Methods). We found that the number of genes that vary significantly in expression level over development in *D. sechellia *remained significantly higher than in both *D. simulans *and the hybrids but it was no longer significantly greater than *D. melanogaster *(χ^2 ^test, 1 df, *P *= 0.02996, 0.0001467, and 0.1891, respectively).

### Between species divergence in the context of ontogeny

**Figure 1:**
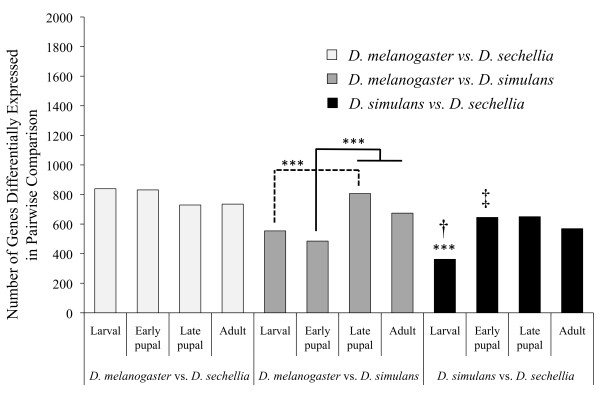
**Number of genes significantly differentially expressed in pairwise comparisons between each of the three pure *Drosophila *species at each of the four sampled stages (out of 2,253 genes)**. There are fewer genes differentially expressed in the larval as compared to the late pupal stage in the comparison between *Drosphila melanogaster *and *D. simulans *(*P *< 0.001) as indicated by the dashed line. Similarly, there are fewer genes differentially expressed in the early pupal stage of the same comparison than the late pupal or the adult as indicated by the solid line (*P *< 0.001). In the comparison between *D. simulans *and *D. sechellia *there are fewer genes differentially expressed during the larval stage than any other stage as indicated by the three asterisks (***) (*P *< 0.001). All other between stage comparisons within the pairwise comparisons were not statistically significant. There were fewer genes that were differentially expressed between *D. simulans *and *D. sechellia *during the larval stage than the other two more phylogenetically distant comparisons (indicated by the dagger [†]). The number of genes that were significantly differentially expressed differed in all three comparisons between the early pupal stage; however, the *D. simulans *versus *D. sechellia *comparison was not the lowest (indicated by the double-dagger [‡]).

Focusing only on the pure species, we compared the number of genes that were differentially expressed at each of the four sampled time points in pairwise comparisons among those genes detectibly expressed at all stages in all three species (2,253 genes; Figure 1). We find that in those pairwise comparisons between species in which the number of differentially expressed genes varied significantly over our sampled developmental interval (*D. simulans *versus *D. melanogaster*, and *D. simulans *versus *D. sechellia*) fewer genes were differentially expressed at the earlier stages (the larval stage in both pairwise comparisons and the early pupal stage in the latter; χ^2 ^test, 1 df, *P *< 0.001). In no case were there significantly fewer genes differentially expressed at a later developmental time in comparison to an earlier one (*P *> 0.05). As *D. simulans *and *D. sechellia *form a clade excluding *D. melanogaster *[20], we expected that they would show fewer differentially expressed genes than the other two possible pairwise comparisons. This was the case in all situations in which the difference between comparisons was statistically significant for the larval, late pupal and adult time points (Figure 1). However, there are significantly more genes differentially expressed between *D. sechellia *and *D. simulans *than in the comparison between *D. melanogaster *and *D. simulans *during the early pupal point (*P *< 0.001 in all cases). We also found significant asymmetry in the number of genes differentially expressed in comparisons between *D. melanogaster *and *D. simulans *or *D. sechellia *during the larval and early pupal time points, where fewer genes are differentially expressed in the former as compared to the latter (*P *< 0.001), suggesting that *D. simulans *and *D. sechellia *have experienced different evolutionary pressures in earlier developmental stages.

### Hybrid expression patterns over development

**Figure 2:**
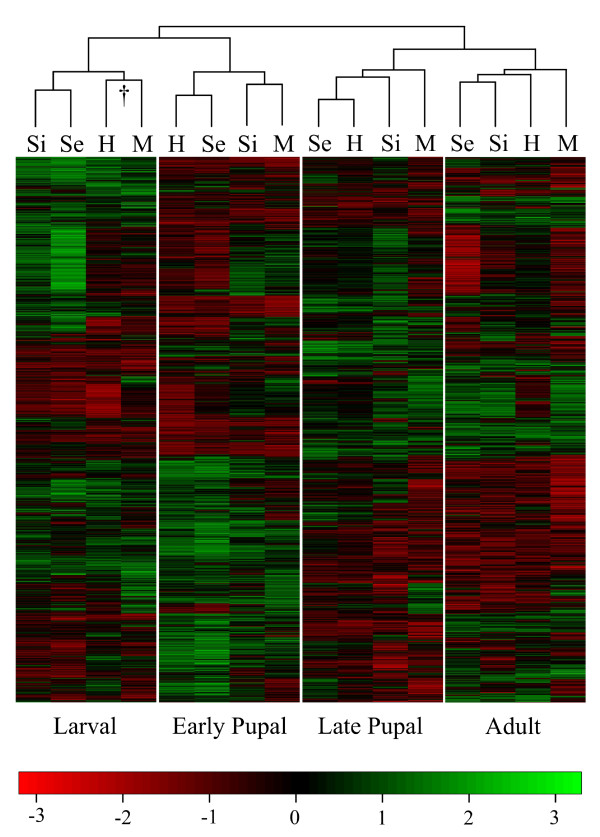
**Hierarchically clustered heatmap indicating relationship between species and stage specific expression patterns for the 2,006 genes detectibly expressed in all three pure species and the hybrids**. (M) *Drosphila melanogaster*; (H) Hybrids; (Se) *D. sechellia*; (Si) *D. simulans*. Clustering relationships were bootstrapped, and all clusters are supported at the 95% level with the exception of the M-H pair indicated by the dagger (†). Genes indicated in red are expressed at a lower level than the reference sample whereas those in green are expressed at a higher level than the reference. All log_2 _transformed expression ratios are scaled between -3 and 3 by the software.

In order to further probe the divergence among regulatory factors between species, we compared patterns of gene expression among the three pure species as well as the male *D. sechellia *and *D. simulans *sterile F1 interspecific hybrids during the four sampled developmental time points. We performed hierarchical clustering analysis using a conservative set of 2,006 genes detectibly expressed on all replicate array spots during all four developmental time points in the three pure species and the hybrids (Figure 2; see Methods). Bootstrapping the dendrogram indicates that all clusters except one are supported at the 95% level (indicated by the dagger [†]). Patterns of expression within individual stages, rather than species, clustered together, indicating that stage-specific expression levels are significantly conserved between species. Furthermore, the larval and early pupal time points formed a cluster, as did the late pupal and adult points, which supports previous observations that suggest that substantial expression turnover occurs during the active organogenesis taking place during the pupal stage [24]. With the exception of the early pupal point, the clustering pattern matched phylogenetic expectations - *D. sechellia *and *D. simulans *cluster to the exclusion of the more distantly related *D. melanogaster *(see also below). The relationship of the hybrids relative to their parental species varied over the sampled developmental interval: in the case of the larval and adult stages, *D. simulans *and *D. sechellia *clustered together to the exclusion of the hybrids, indicating substantial non-additivity of expression levels in the hybrids (note that the clustering of the hybrids with *D. melanogaster *during the larval stage is not supported at the 95% level). In the case of the two pupal time points, however, the hybrids clustered with *D. sechellia*, suggesting that this parent's allelic contribution acts in a manner dominant to that of *D. simulans*.

**Figure 3:**
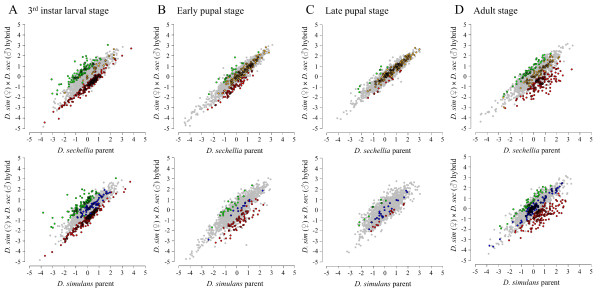
**Scatterplots comparing the log_2_(sample/reference) expression values between each of the parental species (*Drosphila sechellia*, top; *D. simulans*, bottom) versus the F1 hybrids at the (A) larval, (B) early pupal, (C) late pupal and (D) adult stages**. Green: genes overexpressed in the hybrid relative to both parents. Red: genes underexpressed in the hybrid relative to both parents. Orange: genes significantly differentially expressed between parental species and expressed at *D. sechellia *levels in the hybrid. Blue: genes significantly differentially expressed between parental species and expressed at *D. simulans *levels in the hybrid. Grey: all other genes. The number of genes represented in each category is shown in Table 1.

We also compared the number of genes that were differentially expressed in pairwise comparisons between the hybrids and their parental species at each stage among those genes detectibly expressed in all three (2,052 genes; Additional File 5). Contrary to the expectations of the hypothesis that misexpression of genes in the adult stage of hybrids results from a cascade caused by the initial misexpression of a smaller number of genes at an earlier developmental stage [6], the larval stage showed the highest proportion of significantly misexpressed genes (either over- or under-expressed in the hybrid relative to both parents)- 303 - while the fewest (24) were observed during the late pupal time point (the same 2,052 genes are being compared at each time point). Thus, the ontogenic pattern of the proportion of misexpressed genes, from fewest to most, was: late pupal < early pupal < adult < larval (the difference in the proportion of genes that are misexpressed is statistically significant among all pairwise comparisons between stages; χ^2 ^test, 1 df, *P *< 0.001; Figure 3; Table 1). In total, 491 genes were misexpressed during at least one sampled developmental time point, of which 389 (79.2%) were misexpressed only at a single time point. This indicates that misexpression during the larval stage, for instance, is not a good predictor of continued misexpression in subsequent stages as could be predicted by the cascade model, assuming that not only does the proportion of genes misexpressed over time increase but also that genes misexpressed during a particular developmental stage continue to be misexpressed during subsequent stages. Only two genes (FBgn0052652 and FBgn0031920) are misexpressed during all the four time points. Both of these genes are expressed in the testes of *D. melanogaster *[25]. However, their function(s) are unknown, and BLAST searches of the predicted coding sequences from *D. melanogaster *of both genes against the genomes of the three pure species failed to find significant similarity to any genes of known function. Thus, it appears that there is substantial stage-specific autonomy of regulatory breakdown. Interestingly, however, there appears to be an over-representation of male-biased genes (MBGs), or genes with elevated expression in pure species males compared to females, as well as an under-representation of female-biased genes FBGs) among misexpressed genes that are underexpressed in the parents relative to the hybrids during the larval, early pupal and adult time points (χ^2 ^test, 1 df, *P *< 0.001 in all cases). This suggests that, despite such autonomy, there exist predictable commonalities among misexpressed genes [26].

**Table 1:** Number of genes classified into categories based on their patterns of expression in hybrids relative to the parental species.

		Stage		
**Pattern**	**Larval**	**E. pupal**	**L. pupal**	**Adult**

Not differentially expressed	1216	1455	1479	1203
Intermediate expression	0	9	24	8
*Drosphila simulans *dominance	60	13	24	146
*D. sechellia *dominance	27	146	174	83
Underexpressed	206	76	14	149
Overexpressed	97	23	10	53
Other	446	330	327	410

E pupal, early pupal; L pupal, late pupal.

Patterns are as follows: not differentially expressed, not significantly differentially expressed in any pairwise comparison among parental species or hybrids; intermediate expression, expressed at an intermediate level between parental expression levels; *Drosphila simulans *dominance, significantly differentially expressed between parental species and for which hybrids are only significantly different in expression level from *D. sechellia*; *D. sechellia *dominance, opposite of previous pattern; Underexpressed, genes that are significantly underexpressed in the hybrids relative to both parents; Overexpressed, genes that are significantly overexpressed in the hybrids relative to both parents; Other, genes whose expression patterns did not fit into any of the above classes.

**Figure 4:**
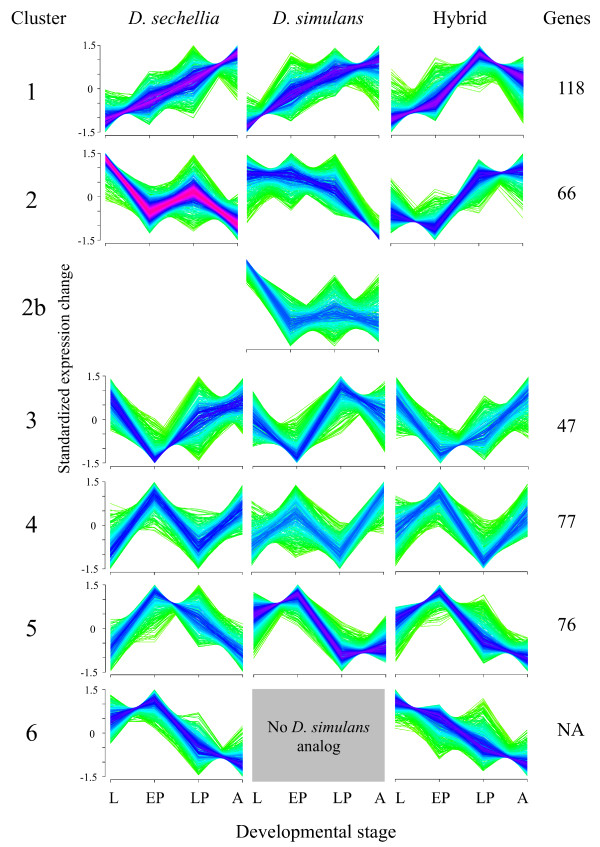
**Fuzzy c-means soft clustering of four sampled stages in hybrids and parental species. Lines from green through blue to red indicate genes that are closer to the cluster centroids**. The six clusters are arranged such that they are horizontally adjacent to the cluster with the largest proportion of shared genes between the species and hybrid. In the case of *Drosphila simulans *two clusters share the majority of their genes with the same cluster in the other two, and are indicated in the order of which shows the highest shared proportion of genes as clusters 2 and 2b. In the case of cluster six, there is no analogous *D. simulans *cluster that shares the majority of its genes with either the *D. sechellia *or hybrid clusters. L, larval; EP, early pupal; LP, late pupal; A, adult.

In order to further dissect the relationship between expression in the hybrid and each of its parents, we compared hybrid expression profiles at each time point to each of the parents individually. We observed no significant differences among time points in the number of genes differentially expressed between *D. simulans *and the hybrid (χ^2 ^test, 3 df, *P *= 0.1322). However, when comparing the *D. sechellia *and the hybrid, both the early and late time points of pupal stage showed significantly fewer differentially expressed genes than either the larval or adult stages, supporting the results of the clustering analysis, which indicates that the hybrids and *D. sechellia *have similar expression profiles during both sampled pupal time points (χ^2 ^test, 1 df, *P *< 2.2 × 10^-16^). The number of genes differing significantly in expression level between the hybrids and either of their parental species' males was significantly different between the two parents (*D. simulans *versus the hybrids compared to *D. sechellia *versus the hybrids; *P *< 0.001): the larval and adult stages showed a greater number of differentially expressed genes in comparison with the hybrids and *D. sechellia*, whereas the early- and late pupal time points showed the opposite pattern. In order to compare the patterns of expression in hybrids relative to their parents in the context of the entire developmental interval, we used fuzzy c-means soft clustering on the same 2,052 genes described above [27], which allowed us to define six clusters which represented major patterns of expression variability over the sampled ontogenic interval (Figure 4). While the majority of clustering patterns are conserved between the parental species, there were several patterns that appeared to be altered in the hybrids (for example, clusters 1, 2 and 3). Of particular interest is cluster 1, which shows an apparent bias towards under-expression during the adult stage in the hybrids and over-representation of MBGs as well as under-representation of FBGs in both parents and the hybrid. In the hybrids, both clusters 1 and 2 also showed a significant paucity of genes known to have lethal mutant phenotypes, which suggests that a bias exists towards greater interspecific conservation of gene regulation among essential genes (see Methods; Additional File 2, sheet E) [26,28].

Another way to assess the similarity between hybrids and their parental species is to determine how the hybrids resemble either parent in terms of the degree to which genes change in expression level between each of our three sampled sequential developmental transitions (larval to early pupal [L to EP], early to late pupal [EP to LP] and late pupal to adult [LP to A]). Thus, we restricted our analysis to those genes that varied significantly in expression level in both parental species and the hybrids at each developmental transition. We then performed linear regressions followed by ANCOVA in order to determine if the hybrids were more similar to one parental species than the other (Additional File 6). Hybrids were significantly more correlated with one of the two parents during the EP to LP and LP to A transitions (F_1298_, F_1436_, and F_1244 _= 3.1602, 19.007, and 112.72, *P *= 0.07647, 1.626 × 10^-5^, < 2.2 × 10^-16^, for the L to EP, EP to LP, and LP to A transitions, respectively). In the case of the EP to LP transition, the degree to which genes change in expression level in the hybrid is more significantly correlated with *D. sechellia *(slope [*m*] = 0.8963, *r*^2 ^= 0.9219) than *D. simulans *(*m *= 1.531, *r*^2 ^= 0.8381). However, during the LP to A transition, the hybrid switches to being more significantly correlated to the *D. simulans *parent (*m *= 1.157, *r*^2 ^= 0.6417) as compared to the *D. sechellia *parent (*m *= 0.6630, *r*^2 ^= 0.8011).

## Discussion

### Consideration of microarray methodology

Previous studies have shown that using single-species microarrays in order to measure the expression levels of the mRNA of multiple species can produce biased estimates due to sequence divergence between the probe and hybridized mRNA if appropriate statistical thresholds are not employed [29]. With this caveat in mind, we sought to minimize the effect of binding bias on our analysis by: (a) employing a minimum 1.5-fold change, expression threshold for expression differences to be considered significant; and (b) analysing only genes with expression information on all available replicate spots. Gilad *et al*. [29] noted that a 1.5-fold threshold difference in the expression level provided near 100% specificity (albeit at a cost in sensitivity) to accurately measuring significant expression differences between human and orangutan mRNA samples hybridized on human microarrays, which differ in nucleotide sequence by approximately 3% (*D. melanogaster *and *D. simulans*/*D. sechellia *differ ~3% [30]). While this has led to a reduction in the size of our dataset which is available for analysis, as well as a reduced sensitivity to significant changes in expression level, the increased specificity is necessary in order to minimize the possibility of spurious inferences. We further validated the results of our expression analysis by testing for a potential correlation between pairwise expression and sequence divergence among the three pure species and found no significant correlation (Spearman rank correlation test, *P *> 0.05) suggesting that our methods were appropriately conservative (see Methods).

However, it should be noted that the use of such conservative methods does impose a cost on the general applicability of the overall analysis, as our final dataset involves the analysis of ~20% of the genes spotted on the *D. melanogaster *12Kv2 cDNA microarray. In order to ascertain the possibility that our final dataset was not representative of the genomic fraction probed by the array, we tested whether those genes analysed showed enrichment, or a paucity, of certain gene ontology (GO) terms as compared to the overall array using the FATIGO web tool [31]. The distribution of GO terms among the genes analysed was not significantly different from that represented on the entire array (data not shown). We also tested for the possibility that genes used in the analysis were more conserved than the array-wide average, as would be expected if the reason that a significant portion of the genes were being rejected was due to binding artifacts generated by probe-sequence divergence. However, we found a statistically significant bias in favour of greater divergence among genes used in the analysis, when compared to those that were not used for all three species (see Methods). While this may suggest that the genes employed in the analysis are, on average, more rapidly diverging than the genes that were excluded from analysis, it does not suggest that our analysis is biased towards genes that show greater sequence conservation because of artifacts resulting from probe/RNA divergence.

Despite this, a certain degree of bias may be expected in our dataset given the limitation of our analysis to genes that were detectibly expressed in all stages among the species being compared. Applying such a restriction avoided the possibility that a gene would be considered not expressed at a particular developmental time-point simply because of technical errors associated with probe/RNA binding on the microarrays. However, it also carries the negative consequence of ignoring genes that are legitimately expressed at very low levels during certain periods of ontogeny. It is unlikely that much useful information was lost during such stages as microarrays are known to have low power to detect significant differences in comparisons between samples with very low absolute expression levels [32]. Conversely, if genes that were excluded from our analysis due to their very low expression levels during particular stages showed significant between-species/hybrid differences in expression levels during other stages, such differences would be missed in our analysis. It is not clear if the value of inclusion of such genes would offset the potential biases introduced by including false-negatives (see above). Such considerations will be resolved as newer, more sensitive, techniques are employed to answer such questions (for example, RNA-seq). In addition, these techniques will also give us the capability of inferring meaningful evolutionary change in expression level among changes below the 1.5-fold expression threshold employed in this study. Nevertheless, a note of caution is warranted in studies that seek to compare loci with minute expression differences: substantial inter-strain variation has been observed in *Drosophila *[33] and, thus, it will be important to determine whether such differences are strain-specific or species-wide.

### Within-species variation in expression levels over ontogeny

Our estimates of the percentage of genes that vary significantly in expression level over the course of ontogeny (~60%-80%) are similar to those reported in a previous analysis of gene expression over the entire course of ontogeny in *D. melanogaster *(~86%) [24]. The reduction in the proportion of genes varying significantly in expression level between stages in our study is most likely a result of our having sampled only the latter portion of development. The significant increase in the proportion of developmentally modulated genes in *D. sechellia *is striking. However, we noted a systematic bias towards low between-replicate variance in *D. sechellia *compared to the other samples. Correcting for this bias in *D. sechellia *caused much of the elevated signal of developmental modulation to disappear. Despite this, the general pattern of an elevated number of developmentally modulated genes in *D. sechellia *remained significant in comparison with *D. simulans *which, again, suggests that *D. sechellia *may have undergone lineage specific divergence in terms of its developmental expression profiles. It is also interesting to note that the observed reduction in between replicate variance in *D. sechellia *is consistent with previous population genetics studies of nucleotide diversity in this species, which have found a significantly reduced level of within species polymorphism relative to *D. simulans *or *D. melanogaster *[20,23] (see below).

### Regulatory divergence in the context of ontogeny

When comparisons between stages were statistically significant, as was the case in the comparisons between *D. melanogaster *and *D. simulans *as well as *D. simulans *and *D. sechellia*, we found that expression level is more conserved between the pure species during earlier as compared to later stages (Figure 1). Thus, Von Baer's [10] classic observation that earlier stages of ontogeny are more conserved than later stages may apply at both the level of nucleotide sequence [14-16] and transcript abundance (see also Additional File 1 for an analysis of nucleotide divergence in the context of ontogeny using the expression data from the present study). Expression levels are not significantly more conserved during earlier developmental stages in our comparison between *D. melanogaster *and *D. sechellia*, which may be the result of two possibilities (Figure 1). First, our analysis may not have provided the sensitivity required to detect differences in the divergence patterns of expression over development in this comparison. Our observation of a reduced level of inter-replicate variability in *D. sechellia *may have led to our observation of a relatively uniform signal of significant divergence in expression level over ontogeny relative to *D. melanogaster*. Alternatively, it is possible that *D. sechellia *has been subject to substantial divergence in expression levels in early developmental stages, more of which are shared with its closer relative *D. simulans*, as compared to *D. melanogaster*. It is possible that, while we are able to detect significant difference among stages in comparisons with *D. simulans*, similar comparisons with *D. melanogaster *show a more uniform distribution of divergence over development.

Von Baer's pattern is not represented in comparisons among the hybrids and either of their parental species (Additional File 5). In the case of the comparison with *D. simulans *there is no significant difference in the proportion of genes differentially expressed among the four sampled developmental time-points, whereas, in the case of the comparison with *D. sechellia*, the intermediate time points show less divergence. Such an observation may indicate that the hybrids neither represent an intermediate phenotype between their parental species, nor are they more closely allied with one over the entire course of development. Rather, the hybrids may express phenotypic traits more consonant with one parent as compared to the other, which varies over the course of ontogeny (see below). This lack of a Von Baerian trajectory in expression level is also apparent among misexpressed genes in the hybrids, where the larval stage shows the highest proportion of misexpressed genes and the two sampled pupal time points the least (note that a gene is only considered 'misexpressed' if it is either under- or over-expressed in the male hybrids relative to both male parents). While this seems to contradict the evidence for a greater conservation of expression levels during earlier stages observed in comparisons of parental species, it is possible that comparisons of expression level among pure species and pure species and their hybrids are measuring two different aspects of regulatory divergence. Whereas interspecific comparisons reveal only the ultimate outcome or regulatory divergence (expression level), comparisons between hybrids and their parents allow us to observe the result of divergence at all levels of gene regulation, from *cis*-regulatory elements to the feedback/forward regulation occurring in the overall hybrid regulatory network. During the larval stage, we may be observing what True and Haag [22] have termed 'developmental systems drift': stabilizing selection appears to be the primary evolutionary force acting upon expression levels for the majority of genes [34], and while the underlying regulatory machinery may have diverged between *D. sechellia *and *D. simulans*, this divergence may be compensatory such that it does not manifest itself in terms of expression level differences between the parental species [35] (Figure 1). Rather it reveals itself only as improper regulation in the case of the hybrids (see below). The larval stage is characterized by a high rate of growth, which is associated with a rapid increase in transcription of total mRNA and translation of proteins [36], which may generate selective pressure in order to maintain uniformly high expression levels despite substantial divergence among underlying regulatory machinery. Such non-adaptive divergence of complex regulatory systems is facilitated in species with reduced effective population sizes, due to the increased probability of fixation of neutral or slightly deleterious mutations [37], which, as noted above, is known to be the case in *D. sechellia*. On the other hand, the complex organogenesis occurring during metamorphosis may involve greater integration among the regulatory circuits (transcription factors or *cis*-regulatory elements) than other stages, leading to its underlying machinery being more refractive to divergence causing misexpression in hybrids. Nevertheless, assuming that an organism's ultimate phenotype determined by gene expression levels (and subsequent post-transcriptional regulation), the conserved expression levels among parental species during earlier developmental stages is probably a compelling validation of Von Baer's third law.

Both our clustering analysis (Figure 2), as well as our analysis of the number of genes differentially expressed in pairwise comparisons, support phylogenetic expectations of between-species divergence in the case of the larval, late pupal, and adult stages (*D. sechellia *and *D. simulans *are more similar to one another). However, this is not the case during the early pupal stage, where *D. melanogaster *and *D. simulans *cluster together and show the fewest significantly differentially expressed genes (Figure 1). This may suggest that, as part of its adaptation to its host plant (*Morinda citrifolia*), *D. sechellia *may have been exposed to unique selective pressures that have altered particular aspects of its larval or early pupal development. This hypothesis is supported by previous studies that have examined developmental phenotypes in the *D. melanogaster *group [38-40] and have found evidence of altered developmental phenotypes in *D. sechellia *relative to other species, where the opportunity for selection may have been responsible for altering aspects of its ontogeny. An alternative explanation for our observation of *D. sechellia*'s status as an outlier is that 'typical' gene expression levels in this species would most probably be observed when it is living on its native host, *M. citrifolia *[41]. By raising our species/hybrids on standard cormeal/molasses/agar medium, we may have induced stress in *D. sechellia*, leading to a pronounced difference in expression patterns relative to the other species. We tested to see if genes that were uniquely differentially expressed at each stage in *D. sechellia *were over-represented in terms of certain GO categories relative to our entire dataset, although no differences were statistically significant (data not shown). The possibility does remain, however, that some proportion of *D. sechellia*'s unique expression patterns results from stress (though these flies have been kept on standard cornmeal medium since the mid-1980s; W. Haerty personal communication), but the present analysis of the hybrids in particular would have been complicated were *D. sechellia *raised on medium containing *M. citrifolia *while *D. simulans *was not.

### The developmental basis of hybrid misregulation

A number of studies involving genome-scale transcriptional profiling have revealed that a substantial proportion of the transcriptome (> 10%) is misexpressed in interspecific hybrids relative to their parental species [6]. Work in the field of speciation has been quite equivocal about the relative number of genes involved in producing the sterile phenotype observed in hybrids. However, recent evidence has supported the notion that sterility in hybrids between closely related species such as those used in the present study is primarily the result of a small number of genes of relatively large effect (≥ 6) [42]. Thus, it appears unlikely that the misexpression observed in our study stems from the incompatible divergence of *cis*-regulatory factors at such a large number of loci. More plausible is the hypothesis that the large scale-patterns of misexpression observed in interspecific hybrids result from divergence of a smaller number of loci having widespread effects in *trans *- perhaps corresponding to some of the loci of large effect revealed through introgression studies [43,44]. However, our study does not support the suggestion that these *trans *acting factors are derived from the cascading effects of a smaller number of genes that are significantly misexpressed at earlier stages of development [6]. In contrast, it would appear that there is considerable stage-specific autonomy of regulatory breakdown, with no obvious pattern of an increasing proportion of genes misexpressed during subsequent stages. MBGs are over-represented among under-expressed, misexpressed genes during three of the sampled developmental stages, indicating that common regulatory factors or selection pressures (for example, sexual selection on MBGs in the adult) may nevertheless underlie misexpression at multiple stages.

The highest proportion of genes is significantly misexpressed during the larval stage (Figure 3; Table 1). Two non-mutually exclusive hypotheses may account for an elevated degree of misexpression during this early stage. First, as mentioned above, the larval stage is characterized by a rapid increase in transcription of total mRNA and translation of proteins [36]. Slight heterochronic changes in hybrid development (for example, later activation of transcriptional machinery) may manifest themselves as widespread under-expression or even over-expression of larval genes. Some evidence for this hypothesis is provided by previous observations which suggested that spermatogenesis may be delayed in third instar larval hybrids between *D. simulans *and *D. mauritiana*, which could lead to qualitative differences in expression pattern of genes involved in this process [44]. Secondly, previous studies have suggested that *D. sechellia *has been subject to divergence in embryonic and larval ontogeny [38,39]. As stabilizing selection appears to be the primary evolutionary force acting upon expression levels for the majority of genes [34], this divergence may not manifest itself in terms of expression level differences in the parental species (Figure 1). Rather, the underlying regulatory machinery may have diverged revealing itself as an elevated proportion of incompatibilities in hybrids (see above).

If we restrict our analysis to genes expressed in the hybrids that are significantly differentially expressed between males of *D. simulans *and *D. sechellia*, they are significantly more likely to be expressed at the *D. simulans *level in the hybrids during the larval and adult stages, whereas they are more likely to be expressed at *D. sechellia *levels during the two pupal stages (Figure 3, Table 1). One may assume that expression levels would generally show an overall dominance in hybrid males in the direction of the parent from which it inherits its X chromosome (in this case *D. simulans*), assuming, of course, that a significant number of regulatory loci are harboured on the X. Our results suggest that this is not the case during all stages and, while the ability of regulatory factors to interact is more conserved during the pupal time points, significant divergence has occurred between the two parental species that manifests itself dominantly with regards to *D. sechellia*. This hypothesis is supported by our observation that the degree to which genes vary in expression level between developmental transitions in hybrids is significantly more similar to the *D. sechellia *parent during the EP to LP transition (Additional File 6).

A classic study, testing the vulnerability of various stages of *Drosophila *ontogeny to induced mortality when exposed to X-rays, found that susceptibility is highest during pupation, which suggests that this stage is particularly sensitive to deleterious perturbation [45]. Such an observation is particularly intriguing in the context of Raff's [12] 'developmental hourglass' hypothesis, which argues that stages involved in organogenesis may be more resistant to evolutionary divergence than preceding or subsequent stages. The original hypothesis focused on embryogenesis. However, our observation of a significantly reduced number of misexpressed genes during the two sampled pupal time points suggests that the mechanisms underlying gene regulation during this stage may be more conserved (Table 1). Holometabolous insects such as *Drosophila*, undergo two rounds of extensive organogenesis (for example, embryogenesis and metamorphosis), and may have two periods of increased regulatory conservation. Interestingly, however, the decreased proportion of genes significantly misexpressed in the hybrids during the pupal stage does not coincide with a decreased proportion of genes significantly differentially expressed between the two parental species (Figure 1).

## Conclusions

In summary, our comparative analysis of transcriptional patterns over the course of ontogeny among species and hybrids of the *D. melanogaster *group has revealed the following major results: (1) in comparisons between the pure species, gene expression levels are more conserved during earlier stages of development as compared to later stages. (2) However, this is not the case in comparisons between parental species and their interspecific hybrids where the mechanisms underlying gene expression appear to be more conserved during the pupal stage suggesting that the underlying regulatory systems are diverging despite the maintenance of expression levels among pure species. (3) There is considerable stage-specific autonomy of regulatory breakdown in hybrids and no obvious pattern of increasing proportion of genes misexpressed over the course of ontogeny, which does not support a cascade model explaining hybrid misexpression. Finally, (4) the number of genes differentially expressed between stages support phylogenetic expectations (that is, are fewer in comparisons between *D. simulans *and *D. sechellia*) for all stages except the early pupal stage. Our findings have implications for the fields of both evo-devo and speciation. First, they support the extension of Von Baer's [10] 'third law', or the more modern developmental hourglass hypothesis, to the level of the transcriptome, lending support to previous observations which suggested that similar forces may act to limit both gene expression levels and coding sequence divergence [27,28]. Secondly, while it has already been remarked that the widespread misexpression of genes observed in interspecific hybrids is unlikely to be the result of equally widespread divergence of *cis *regulatory elements [6], our results suggest that regulatory factors (for example, proteins, mRNAs) experience stage-specific, autonomous incompatibilities, leading to similarly stage-specific patterns of misexpression. Several of the so-called 'speciation' genes (loci that contribute to hybrid dysfunctions such as sterility or inviability) that have been identified are predicted to have transcription factor activity and regulate expression of downstream genes in *trans *[6]. The findings presented here suggest that a more complete understanding of stage-specific gene regulatory networks, which would enable us to identify those nodes that may ultimately control the suite of genes identified as misexpressed in hybrids, may be a fruitful approach to identifying new loci underlying both developmental evolution, reproductive isolation, and ultimately speciation.

## Methods

### Collection of *Drosophila *and microarray hybridization

Time synchronized, stage-specific collection of *D. melanogaster *(14021-0231.00), *D. sechellia *(Cousin Island, Jean R. David, Centre National de la Recherche Scientifique, Gif sur Yvette, France), *D. simulans *(14021-0251.2) and the *D. simulans *(♀) × *D. sechellia *(♂) F1 hybrid individuals was performed according to the protocol described in Additional File 1 at the following time-points: 3rd instar larva (96 h post larval eclosion), early pupal (2 h post-puparium formation [ppf]), late pupal (72 h ppf), and adult (1.5 h post adult eclosion). mRNA was extracted from 25 males from each stage and species/hybrid using the RNeasy Mini kit (Qiagen, Hilden, Germany). Given that it was impractical to collect sufficient mRNA from hybrids for direct use in microarray hybridizations, all mRNA samples were then amplified twice using the MessageAmp II aRNA kit (Ambion, Texas, USA). A much larger amount of *D. melanogaster *mRNA was extracted from each stage in order to use as an equal concentration mixed-stage (unamplified) reference on the cDNA microarrays. All samples, as well as the reference, were sent to the Canadian Drosophila Microarray Centre (CDMC, http://www.flyarrays.com) for hybridization on *D. melanogaster *12Kv2 cDNA microarrays spotted with ~12,000 elements representing approximately 10,000 unique genes. In the case of *D. melanogaster*, *D. sechellia*, and *D. simulans*, the amplified mRNA from a single pool of 25 male flies was hybridized on three technical replicate microarrays and analysed according to the protocols provided in Additional File 1. However, in the case of the hybrids, mRNA from three separately extracted and amplified pools of 25 males were each used for hybridization to a single microarray, such that the replicates were also biological as well as technical, in order to determine whether there was a significant effect of between-extraction/amplification variability on our estimates of expression differences. We found no significant increase in variability among biological replicates indicating that pools of 25 individuals captured the majority of biological variability (Additional File 1). Both raw and normalized expression values for all arrays are deposited in the Gene Expression Omnibus http://www.ncbi.nlm.nih.gov/geo/ under accession number GSE17535.

### Clustering analyses

The average log_2_(sample/reference) ratios among all replicate spots within a stage were collected for the 2,006 genes detectibly expressed in all species/hybrids at all stages and used in order to draw a heatmap showing a hierarchical clustering among stages using the 'Heatplus' package in Bioconductor [46]. The pairwise dissimilarity matrix used in the clustering was generated using the Spearman coefficients of correlation of the log_2_(sample/reference) ratios between stages, under the hclust() function using the 'complete' method. The clustered dendrogram was then bootstrapped using the 'pvclust' package in R [47] with 2,000 bootstrap replicates. Time-course clustering was performed using the 2,053 genes detectibly expressed at all four stages in the hybrids and their two parental species using the 'Mfuzz' package in Bioconductor [26]. The cselection() function was used to determine that 6 clusters and an m value of 2.5 produced both distinct clustering patterns and no empty clusters in any of the pure species or hybrids.

### Analysis of sex-biased and 'essential' genes

In order to determine patterns sex-bias in expression, we obtained the *D. melanogaster *based data from the Sebida database [48]http://141.61.102.16:8080/sebida/content/download/sebida_melanogaster.txt of which 2,037 of the 2,052 genes detectably expressed in *D. sechellia*, *D. simulans *and the hybrids (Additional File 2, Sheet A) were represented. The direction of sex-bias, if any, was obtained from the 'MetaClass' column of the dataset, which represents a concatenated list of sex-biased genes derived from multiple previous datasets. The number of genes falling into each class of bias, male-biased (MBG), female-biased (FBG), and unbiased (UBG), in each category (for example, in a cluster from Figure 4, or misexpressed in the hybrids, see Results) was compared against the distribution represented in all genes being analysed using Bonferroni corrected χ^2 ^tests in order to determine if a particular class was over/under-represented in a category of genes (Additional File 2, Sheet E). 'Essential' genes were determined by pooling all *D. melanogaster *genes with known lethal mutant phenotypes as determined from FlyBase release 2009_6 (7 July 2009), representing 894 genes in our total dataset (Additional File 2, Sheet A). As above, the number of genes falling into the essential and non-essential classes in each category were compared to the total distribution represented among all genes being analysed using Bonferroni corrected χ^2 ^tests in order to determine if a particular class was over/under-represented in a category of genes (Additional File 2, Sheet E).

### Testing for bias in expression level estimates due to sequence divergence

As previous studies have indicated that sequence divergence between the spotted microarray probes and the mRNA being used for hybridization can lead to biased expression estimates in the absence of appropriately conservative statistical thresholds [29], we tested for a correlation between sequence divergence between the *D. melanogaster *expressed sequence tags (ESTs) spotted on the microarray and *D. simulans*/*D. sechellia *and absolute fold difference in between species expression difference estimates. We obtained the full-length EST sequences of the clones spotted on the CDMC *Drosophila *12K version 2 cDNA microarray from FlyBase for all of the genes in our dataset that were represented in the *Drosophila *12 Genome Consortium *D. melanogaster *group data, representing 1,311 genes [49]. Furthermore, we obtained the longest predicted coding sequence from each of these genes for *D. melanogaster*, *D. sechellia*, and *D. simulans *from the same dataset [49] and performed pairwise alignments between the ESTs and each of the pure species for each gene using Dialign-TX version 1.0.2 [50]. We then calculated proportional-integrated derivative (PID) according to method No. 4 in Raghava and Barton [51], where PID is calculated as the number of identical residues among aligned residues and internal gaps of the shortest aligned sequence: by ignoring gaps outside of the shortest sequence, we also ignore sequence that would not be bound to the probe. As the EST sequence was not always 100% identical to the *D. melanogaster *sequence, for the purposes of calculating between species identity, PID was calculated as the absolute difference between *D. melanogaster *versus EST sequence PID and *D. sechellia *or *D. simulans *versus EST sequence PID (PIDs are reported in Supporting Information File 2A). The absolute fold difference in expression level between *D. melanogaster *and the other two species was obtained as the absolute fold expression difference of the average Log_2 _ratio among all six replicates of the gene in each species at each stage. If sequence divergence led to exaggerated estimates of expression divergence due to differences in array binding kinetics, we would expect to see a negative correlation between absolute fold expression difference between *D. melanogaster *and either of the other two pure species' percent identity. No significant correlation was observed in either pairwise comparison at any of the four sampled developmental time points (Spearman's rank correlation test, *P *> 0.05).

GO analysis was conducted by comparing the subsets of interest (for example, genes uniquely differentially expressed in *D. echolalia*) against the total dataset of analysed genes (for example, genes in common among the three pure species) using FATIGO [31]http://babelomics.bioinfo.cipf.es/ using two-tailed tests, multiple test correction, and retaining any duplicates between lists.

### Comparison of the between replicate variance among expression level estimates

The variance (σ^2^) was estimated for the distribution of replicates spots (within or between arrays) within each stage and for each species/hybrid and the distribution of variances were compared to one another using pair wise, permuted Kruskal-Wallis tests (Additional File 4). In no case was the mean σ^2 ^of the *D. simulans *(♀) × *D. sechellia *(♂) F1 hybrids, which were generated using biological replicate arrays, significantly higher than all pure species (it was always significantly lower than *D. melanogaster*). This confirmed that the majority of biological variability in expression levels was captured in our pools of 25 males. σ^2 ^estimates in *D. sechellia *were significantly lower than all other species/hybrids in the case of the larval, early pupal and late pupal stages (Kruskal-Wallis rank sum test, *P *< 2.2 × 10^-16 ^in all cases). We therefore simulated *D. sechellia *expression data such that *D. sechellia *means would be maintained, but variances would be scaled to *D. simulans *levels using a custom PERL script. The mean log_2_(sample/reference) expression value in *D. sechellia *(), as well the *D. simulans *σ^2 ^(σ^2^_*D. sim*_) for each gene among the 2,006 genes detectibly expressed in all stages in the three species and the hybrids was obtained. Three random numbers, *y*_1_, *y*_2_, and *y*_3_, were then chosen such that they summed to (*n*-1)/2 × σ^2^_*D. sim *_(where *n *= 6 replicate spots). The new distribution of *D. sechellia *values was generated by creating three pairs of values, one for each of the random numbers, each equal to  and . The six simulated replicate array values were then used in order to reanalyze the *D. sechellia *data.

### Statistical analysis

All statistical analyses were performed using the R statistical package version 2.7.2 [52]. Permuted Kruskal-Wallis rank sum tests were performed with 10,000 permutations of the data using the 'coin' package. Permuted 95% confidence estimates were generated using the 'boot' package on 10,000 permutations of the data.

#### Data deposition

All array data have been deposited in the Gene Expression Omnibus under study accession number GSE17535 http://www.ncbi.nlm.nih.gov/geo/query/acc.cgi?acc=GSE17535.

## Abbreviations

CDMC: Canadian Drosophila Microarray Centre; df: degree of freedom; EP: early pupal stage; EST: expressed sequence tag; FBG: female-biased gene; GO: gene ontology; L: larval stage; LP: late pupal stage; MBG: male-biased gene; MRCA: most recent common ancestor; MYA: million years ago; PID: percent identity; ppf: post-puparium formation; UBG: unbiased gene.

## Authors' contributions

CGA and RSS conceived of the study and drafted the manuscript. CGA collected tissues for analysis, extracted and amplified RNA and carried out the data analysis and interpretation.

## Supplementary Material

Additional file 1 **Supplementary methods and analysis**. This supplementary document contains additional analyses and detailed methods that were beyond the scope of the primary manuscript. Click here for file

Additional file 2 **Raw data and results of statistical analyses**. This supplementary document contains tables of all raw data and detailed results of statistical analyses described in the manuscript and supplementary methods and analysis. Click here for file

Additional file 3 **Supplementary figure 1**. Venn diagrams indicating the number of genes varying significantly in expression level over the four sampled developmental stages that are shared among the three pure species and the two parental species and the hybrids. Click here for file

Additional file 4 **Supplementary Figure 2**. Boxplots comparing the distribution of between microarray spot replicate variances for each stage within the three pure species and the *Drosphila simulans *(male) × *D. sechellia *(female) F1 hybrids. Click here for file

Additional file 5 **Supplementary Figure 3**. Number of genes significantly differentially expressed in pairwise comparisons between *Drosphila simulans*, *D. sechellia*, and the *D. simulans *(male) × *D. sechellia *(female) F1 hybrids (*D. sim *male × *D. sec *femalw) at each of the four sampled stages (out of 2052 genes). Click here for file

Additional file 6 **Supplementary Figure 4**. Scatter plots comparing the fold change in expression level between the hybrids and the two parental species for each of the three consecutive developmental transitions. Click here for file
