# Supplementary material for: Molecular evidence for increased regulatory conservation during metamorphosis, and against deleterious cascading effects of hybrid breakdown in Drosophila
Source: BMC Biol. 2010 Mar 31;8:26. doi: 10.1186/1741-7007-8-26 (PMC2907589; doi:10.1186/1741-7007-8-26)
Supplement: Additional file 1 — Supplementary methods and analysis. This supplementary document contains additional analyses and detailed methods that were beyond the scope of the primary manuscript. [file 1741-7007-8-26-S1.PDF]

Additional data file 1. Supplementary Methods and Analysis.

This supplementary document contains additional analyses and detailed methods that were beyond the scope of the primary manuscript. In **Section 1** we present an analysis of the rates of coding sequence divergence of genes classified into specific developmental stages in order to test the conclusions found by Artieri et al. [1]. **Section 2** describes the protocol used to collect the time-synchronized *Drosophila* males for subsequent mRNA extraction. Finally, **Section 3** details the hybridization and analysis protocol used in order to collect our cDNA microarray data.

Section 1: Analysis of Rates of Coding Sequence Divergence over Sampled Developmental Stages.

Artieri et al. [1] found a significant increase in the mean rate of divergence among genes expressed in subsequent stages of *D. melanogaster* ontogeny, classified by the stage at which they showed their highest proportion of representation in stage-specific EST libraries. However, their study was unable to separate genes expressed in the larval stage from those expressed during the pupal stage due to the lack of larval and pupal specific EST libraries. In order to address the dynamics of gene divergence among genes expressed during these stages, we obtained Phylogenetic Analysis by Maximum Likelihood (PAML) Model 0 (a single rate for the entire tree) estimated rates of divergence (d_N, d_S, and d_N/d_S) for all genes in our dataset represented in the *Drosophila* 12 Genomes Consortium [2] *D. melanogaster* group data (representing a total of 1,334 genes). Each gene was classified into 1 of the 4 sampled developmental time points (larval, early pupal, late pupal, or adult) based on the time during which it showed its highest level of expression. The entire dataset of genes was then re-classified into the

same time points using arbitrarily chosen specificity thresholds such that in order for a gene to be classified as specific to a stage, its highest level of expression had to occur at that stage and exceed the next highest level of expression measured at any other stage by more than a 0.25 fold or 0.5 fold expression difference (Additional data file 2, sheet A).

While the comparisons between genes classified into specific stages using no specificity threshold revealed statistically significant differences (see below; Additional data file 2, sheets B and C) very few comparisons remained significant even at low specificity thresholds, likely owing to the much reduced sizes of the datasets. Consequently, we present only the results of the analysis using no specificity threshold, though all results of the analysis performed using a greater than 0.25, or 0.5 fold threshold can be found in Additional data file 2, sheet C. Like Artieri et al. [1], in *D. melanogaster*, we find that when comparisons among divergence rates (d_N and d_N/d_S) between stages are statistically significant, later stages diverge more rapidly than earlier stages (Kruskal-Wallis rank sum test, $P < 0.05$ for the comparisons between either the late pupal or adult stages with the larval stage); the late pupal stage also diverges more rapidly than the early pupal stage in terms of d_N/d_S ($P = 0.049$) (Additional data file 2, sheet C). In terms of d_S, the only significant comparison indicated that the synonymous rate of substitution was lower in the larval stage as compared to the early pupal stage, possibly lending weak support to the findings of previous studies suggesting that codon usage bias is highest during the larval stage [3]. In *D. sechellia*, the only statistically significant comparison in terms of d_N and d_N/d_S indicates that the adult stage is diverging more rapidly than the early pupal stage ($P < 2.2 \times 10^{-16}$); the adult stage is also diverging more rapidly than the late pupal stage in d_N ($P = 0.040$). With regard to d_S, the late pupal

stage appears to be more conserved than the larval and early pupal stages. Interestingly, all comparisons between stages in *D. simulans* were non-significant. Finally, in the case of the hybrid, we found that the late pupal stage diverges more rapidly (d_N and d_N/d_S) than the other three stages, while all other comparisons were non-significant, with the exception of the early pupal stage, which is more conserved than the late pupal stages with regards to d_S (Additional data file 2, sheet C).

Differences between species in the patterns of divergence rates over ontogeny raises the possibility that the stage of highest gene expression may vary significantly between species, especially in comparisons over large evolutionary distances [1]. In order to address this, we compared the proportion of genes that retained the same stage of highest expression in comparisons between *D. melanogaster* and *D. simulans*, as well as *D. sechellia* and *D. simulans* at no specificity threshold (1,334 genes), and a greater than 0.25 or 0.5 fold expression level threshold (212 and 93 genes, respectively). Using no specificity threshold, we find that 53% of genes (708 genes) retain the same stage of highest level of expression in *D. melanogaster* and *D. simulans*, while 55% of genes (732 genes) retain the same stage of highest level of expression in *D. sechellia* and *D. simulans*. Using a greater than 0.25 fold threshold of expression, these numbers become 63% (134 genes) and 76% (161 genes) respectively, a statistically significant increase in the case of the *D. sechellia/D. simulans* comparison (χ^2 test, 1 df, P = 0.00433). Finally when comparing patterns classified at a 0.5 fold or greater specificity threshold, 98% of genes (91/93) retained the same highest stage of expression in both pairwise comparisons, which is a statistically significant increase in conservation of stage of highest expression level as compared to using no specificity threshold in both

comparisons (χ^2 test, 1 df, P < 0.001). These results suggest that the more specific a gene's expression is to a given stage, the more likely it is to retain such specificity in evolutionary comparisons.

Our results generally support those of Artieri et al. [1] in that in *D. melanogaster*, when the differences are statistically significant, later stages diverge more rapidly than earlier stages (Additional data file 2, sheets B and C). This pattern also holds in *D. sechellia* in that statistically significant differences in evolutionary rates are in the direction of more rapid divergence in later stages. In the case of the hybrids, the late pupal time point appears to be the most rapidly diverging in terms of both d_N and d_N/d_S. Interestingly, significantly more genes expressed at their highest level in the adult stage of *D. sechellia* are expressed at their highest level during the late pupal stage in hybrids in comparison to the larval or early pupal stages (χ^2 test, 1 df, P < 0.001). Furthermore, the proportion of genes expressed at their highest level in adults in *D. sechellia* was not significantly different in comparison between genes expressed at their highest level in the late pupal or adult stage of the hybrids, perhaps indicating that a significant portion of genes expressed at an earlier stage in the hybrids as compared to *D. sechellia*, thus leading to a signal of elevated d_N and d_N/d_S in the late pupal stage. However, without a larger dataset of genes such possibilities must be considered purely speculative.

Another significant result of Artieri et al.'s [1] analysis was the observation of a positive correlation between the rate of divergence of genes and the degree to which those genes were specific in the stage at which they were expressed. We were unable to test the results of their analysis in our interspecific comparisons due to the reduced sample-sizes of genes classified into specific stages at even low (i.e., greater than 0.25

higher expression than any other stage) specificity thresholds. However, the ability to repeat such comparisons using a larger data set would be useful, given our observation that genes that are more specific in expression to a given developmental stage are more likely to be conserved in terms of stage of highest expression level between species, and thus would likely provide a more adequate form of comparison of divergence rates over ontogeny between species. Failure to apply adequate specificity thresholds will likely add noise to the analysis resulting from normal intraspecific or intraindividual variance in expression levels, which may explain why no significant differences were observed in comparisons between stages in *D. simulans*.

In addition to evolutionary patterns relating to the stage at which genes show their highest level of expression, genes that vary significantly in expression level during development may be expected to be subject to different evolutionary forces as compared to those whose expression remains stable (Additional data file 2, sheet D). We find that in all 3 pure species as well as the hybrids, genes that vary significantly in expression level diverge less rapidly in terms of d_N than those genes that vary significantly (permuted Kruskal-Wallis rank sum test, $P < 0.05$). This pattern also holds for d_S ($P < 0.001$) except in the case of *D. sechellia* ($P = 1.00$). Mirroring this pattern, the distribution of values of d_N/d_S is significantly greater in genes that vary significantly over development as compared to those that do not vary only in *D. sechellia* ($p = 0.0102$); there are no statistically significant differences in the other 3 comparisons (Additional data file 2, sheet D). Given that in *D. melanogaster*, *D. simulans*, and the hybrids, both the non-synonymous and synonymous rates of substitution, but not their ratio, are elevated in invariant genes, the most likely explanation is relaxation of selective constraint upon

genes that are not modulated in expression level over development. The elevated d_N/d_S observed in *D. sechellia* among genes that do not vary over development may suggest the action of selection; however, given our observation of a significantly reduced between replicate variance in this species, likely leading to an overestimate of the proportion of genes that vary over the course of development, we cannot rule out the possibility that *D. sechellia's* unique pattern is simply an artifact of low within-species expression diversity.

Section 2: Time-synchronized collection of *Drosophila*

Approximately 30 individuals of each species (*D. melanogaster*, *D. sechellia*, and *D. simulans*), both males and females, were placed overnight at 25˚C on a 10 cm plastic petri dish, which was approximately half-full of standard cornmeal – molasses – agar medium upon which a small amount of yeast mixed with lukewarm tap water was painted with a small paintbrush. Each dish was covered by a 100 ml tri-cornered beaker into which small holes had been punctured to assure adequate ventilation while preventing escape of the flies. In the morning, the flies were transferred to a fresh yeast-painted petri dish, while all larvae on the dish from the previous day, visible by inspection under a dissecting microscope, were removed using a syringe. The dish was then allowed to incubate again at 25˚C for 2 hours at which point all newly emerged larvae were transferred onto fresh cornmeal-molasses - agar dishes and placed at 25˚C for 96 hours. No more than 30 individuals were placed on a single plate in order to prevent density dependent growth effects. At 96 hours, during the 3rd instar larval stage, individuals were sexed under a dissecting microscope based on the morphology of the developing gonads [4], and males were anesthetized on ice in RNALater (Ambion) and placed at -80˚C until mRNA extraction (see below).

After 200 individuals of each species had been collected, a similar protocol was followed except that in this case after 30 individuals of each species were placed overnight at 25˚C on a 10 cm plastic petri dish, all larvae found in the morning were transferred to fresh cornmeal – molasses –agar petri dishes, again with no more than 30 individuals per dish, and allowed to develop at 25˚C for approximately 120 hours. Dishes were then examined for the presence of pupae, which were discarded. After 2 hours, any larvae that had begun to undergo pupation (as defined by the presence of a brown puparium and complete lack of movement) were sexed and transferred to fresh petri dishes and allowed to develop for 2 (early pupal time point) or 72 (late pupal time point, post red eye) more hours before collection of males on ice in RNALater and immediate transfer to -80˚C until mRNA extraction. Virgin adults were collected by tapping out pupae-containing jars in the morning, and collecting any flies that had emerged in the following 1.5 hours. Individuals were sexed based on genital morphology, anesthetized on ice in RNALater and transferred immediately to -80˚C until mRNA extraction.

Collection of hybrids

Approximately 10 4-day old *D. simulans* (14021-0251.2) females were placed with 15 newly emerged *D. sechellia* (Cousin Island, Jean R. David Centre National de la Recherche Scientifique, Gif sur Yvette, France) males on petri dishes following the protocol described above. However, unlike in the case of the pure species, newly emerged F1 hybrid flies were never discarded, but rather placed into a separate glass vial with cornmeal-molasses-agar medium and kept for approximately 20 days. Given that the F1 hybrid males from this cross are sterile [5], any larvae observed in the vial after 20

days indicated that at least one the parental females was not a virgin, and thus all individuals collected from that cross were discarded.

Section 3: Microarray Hybridization/Analysis Protocol

In the case of the amplified samples, the following reverse transcription protocol was used: RNase-free water was added to 5 μg of total RNA from each sample to bring the final volume to 14.5 μl. 4 μl of random primer was added followed by incubation at 70°C for 10 min, then 42°C for 5 min. 19.5 μl of modified Indirect RT master mix was added to each tube, along with 2.0 μl of Superscript II RT (Invitrogen) and the reaction was incubated at 42°C for 3 hours. For the unamplified mixed-stage *D. melanogaster* reference sample, the following reverse transcription protocol was used: RNase-free water was added to 60 μg of total RNA from each sample, to bring the final volume to 19 μl. 21 μl of Indirect RT master mix was added into each tube and the reaction was incubated at 65°C for 5 min, then 42°C for 5 min. 2 μl of Superscript II RT (Invitrogen) was added to the sample, followed by incubation at 42°C for 3 hours.

The cDNA product was cleaned and precipitated by adding 8 μl of 1N NaOH to each reaction with mixing by pipetting followed by a quick spin and immediate incubation at 65°C for 10 min. 8 μl of 1N HCl was then added, followed by 4 μL of 1M Tris (pH 7.5), mixing by pipetting after each addition. 38 μl of water was added to bring the total volume to 100 μl, and the amino allyl-cDNA was purified using either the Qiagen PCR clean up or Invitrogen Purelink purification kit (using 80% EtOH for the wash buffer and eluting with 2 × 50 μl of water). After purification, 10 μl of 3M NaOAc, 1 μl of glycogen (20 μg/μl) and 120 μl of ice-cold isopropanol were added, and the

cDNA was allowed to precipitate at –20°C for at least 75 min or overnight. The precipitated cDNA was then spun at >12,000 g for 30 min and the pellet was washed with 200 μl of 75% EtOH, followed by another spin at >12,000 g for 5 min. All EtOH was carefully pipetted from the tube and the probe pellet was allowed to dry for ≤ 1 min before resuspension in 5 μl of water.

Samples were then dye conjugated by addition of 3 μl of 0.3 M $NaHCO_3$ to the resuspended amino allyl-cDNA, followed by 2 μl of reactive dye (Alexa647 for samples, and Alexa555 for the reference) and subsequent incubation at room temperature in the dark for 1 hour. 90 μl of ddH_2O was added to each sample, followed by purification using either the Qiagen PCR clean up or Invitrogen Purelink purification kit, washing with 80% EtOH 3 × and eluting with 3 × 50 μl of water. 15 μl of 3M NaOAc, 1.5 μl of glycogen (20 μg/μl) and 170 μl of ice-cold isopropanol were added to the labeled probe, and the DNA was allowed to precipitate at –20°C for at least 30 min. The precipitated probe was then spun at >12,000 g for 30 min and the pellet was washed with 200 μl of 75% EtOH, and spun at >12,000 g for 5 min. All EtOH was carefully pipetted from the tube and the probe pellet was allowed to dry for ≤ 1 min before resuspension in 5 μl of water.

Microarrays were competitively hybridized using the following protocol: 80 μL of hybridization buffer (75 μl of DIG Easy Hyb [Roche], 4 μl of 10 mg/ml yeast tRNA [Invitrogen], and 4 μl of 10 mg/ml salmon sperm DNA [Sigma]) was added to each resuspended probe, followed by incubation at 65°C for 10 min. Both sample and reference probes were placed on the array, which was then placed in a sealed chamber in a 37°C water bath for 16-18 hours. The array was then washed for 3 × 15 min in pre-

warmed 1 × SSC, 0.1% SDS. The array was then washed with room temperature 1 × SSC for ≤1min, followed by room temperature 0.1 × SSC for ≤ 15 sec. Arrays were scanned using a ScanArray 4000 XL (GSI Lumonics/Packard Biochips); images were preprocessed and quantified using QuantArray v3.0 (PerkinElmer).

Analysis of microarray data

Data from the scanned microarrays were uploaded into GeneTraffic™ DUO version 3.0 (Iobion Informatics) and replicate spots were filtered such that any element showing a coefficient of variation greater than 200% among replicates, or a 2-fold or greater difference among the highest and lowest measured replicate spot (including all elements within an array or between replicate arrays) was flagged. All elements flagged according to these criteria as well as those flagged by the internal quality control standards of the software were then manually inspected for the presence of unacceptable spots (e.g., incorrectly printed, contained visible surface scratches or matter interfering with the scanning, etc.). Such spots were removed, and if less than 6 usable replicates (i.e., 2 replicate spots per array) remained, the entire element was discarded. Furthermore, an element was discarded if a subset of the within-array replicate spots showed consistently different hybridization intensities; these spots likely represent clones that were incorrectly annotated as belonging to the same element in the CDMC's *Drosophila* 12kv2 microarray annotation file (http://142.150.8.217/GT_annot.zip). The filtered raw data was then downloaded from GeneTraffic™ DUO version 3.0 and subjected to a second round of quality control, where spots were removed if they did not show an expression intensity of at least 100, as well as a two-fold expression intensity above either the local or global average background. All genes that did not have usable data

from both replicate spots on all three microarrays in all four stages within a species were then removed from further analysis. The CDMC's *Drosophila* 12kv2 microarray annotation file was then manually inspected in order to identify all spots for which a single Flybase gene number (FBgn) (FB2008_10 Dmel Release 5.13; http://flybase.org/) could unambiguously be identified (the manually updated array spot number/clone - FBgn associations are listed in Additional data file 2, sheet F). All control spots as well ambiguous clone spots were removed from further analysis, leaving only genes that were detectibly expressed in all 4 stages in at least one of the 3 species or the hybrid.

Normalization and microarray data analysis

The data remaining after quality control were normalized using the 'rlowess' procedure (spatial-intensity joint loess) as implemented in the 'maanova' package in the R statistical software (http://research.jax.org/faculty/churchill/software/Rmaanova/index.html) [6], using default values and a 'TwoColor' array type. The output for each spot on each array from maanova was then transformed into its $\log_2$(sample/reference) ratio, which was then analyzed by the software Significance Analysis of Microarrays as implemented in the 'samr' package in R (http://www-stat.stanford.edu/~tibs/SAM/) [7,8]. Differential expression of genes between samples was estimated using two methods: for within-species, between-stage comparisons (i.e., do genes vary significantly in expression over the course of development within a species?), samr was run on all genes from the dataset of 4,286 that were detectibly expressed within a species/hybrid using the 'Multiclass' response type, 2000 permutations of the data, and a False-Discovery Rate (FDR) of 5%.

Furthermore, pairwise, Bonferroni-corrected t-tests were performed on the distribution of replicate values for each developmental transition (larval to early pupal, early pupal to late pupal, and late pupal to adult) for all genes that were identified as varying significantly among the 4 developmental stages. Genes that did not vary significantly (as identified by the t-test) by at least a 1.5 fold expression difference in at least one of the three developmental transitions were rejected as not varying significantly over development. In the case of between pure species or between hybrids and their parental species, within-stage comparisons (i.e., are genes significantly differentially expressed between species/hybrids within a given stage?), samr was run on all genes from the dataset that were detectibly expressed in both species of a given pairwise comparison using the 'Two class unpaired' response type, a minimum-fold expression difference threshold of 1.5, 2000 permutations of the data, and an FDR of 5%. After samr analysis, all remaining genes in the analysis were inspected for duplicates (some genes are spotted in duplicate on the microarray). If a given gene showed the same pattern of expression in all of its duplicates, only the first spot in numerical order according to the annotation file, or the first spot with statistically significant differences, was retained for further analysis. Any duplicates that did not agree in expression pattern (e.g., higher in one species in one duplicate, but higher in the other species in another duplicate) were excluded from further analysis. A summary of all raw data with results of the analysis for the 4,286 genes retained in the final analysis is found in Additional data file 2, sheet A.

REFERENCES

1. Artieri CG, Haerty W, Singh RS: Ontogeny and Phylogeny: molecular signatures of selection, constraint, and temporal pleiotropy in the development of Drosophila. BMC Biol 2009, 7:42.
2. Drosophila 12 Genomes Consortium: Evolution of genes and genomes on the Drosophila phylogeny. Nature 2007, 450:203-221.
3. Vicario S, Mason CE, White KP, Powell JR: Developmental stage and level of codon usage bias in Drosophila. Mol Biol Evol 2008, 25:2269-2277.
4. Ashburner M: Drosophila A Laboratory Manual. Cold Spring Harbor: Cold Spring Harbor Laboratory Press; 1989.
5. Bock IR: Interspecific hybridization in the genus Drosophia. In Evolutionary Biology. Edited by Hecht M, Wallace B. New York: Plenum Publishing Corporation; 1984:41-70.
6. Wu H, Kerr MK, Cui X, Churchill GA: MAANOVA: A software package for the analysis of spotted cDNA microarray experiments. In The Analysis of Gene Expression Data: Methods and Software. Edited by Parmigiani G, Garett ES, Irizarry RA, Zeger SL. New York: Springer; 2003:313-340.
7. Tusher VG, Tibshirani R, Chu G: Significance analysis of microarrays applied to the ionizing radiation response. Proc Natl Acad Sci USA 2001, 98:5116-5121.
8. Storey JD, Tibshirani R: Statistical methods for identifying differentially expressed genes in DNA microarrays. Methods Mol Biol 2003, 224:149-157.
