# Supplementary material for: Molecular evidence for increased regulatory conservation during metamorphosis, and against deleterious cascading effects of hybrid breakdown in Drosophila
Source: BMC Biol. 2010 Mar 31;8:26. doi: 10.1186/1741-7007-8-26 (PMC2907589; doi:10.1186/1741-7007-8-26)

Additional data file 3. Supplementary figure 1 - Venn diagrams indicating the number of genes varying significantly in expression level over the 4 sampled developmental stages that are shared among (**A**) the three pure species and (**B**) the two parental species and the hybrids. The number of genes indicated in each segment of each the diagram is drawn from the 2,006 genes that were detectably expressed in all 3 species and the hybrid at all 4 sampled developmental stages. The numbers under each species' name indicates the total number of genes that vary significantly in expression level over the sampled developmental stages in that species/hybrid.

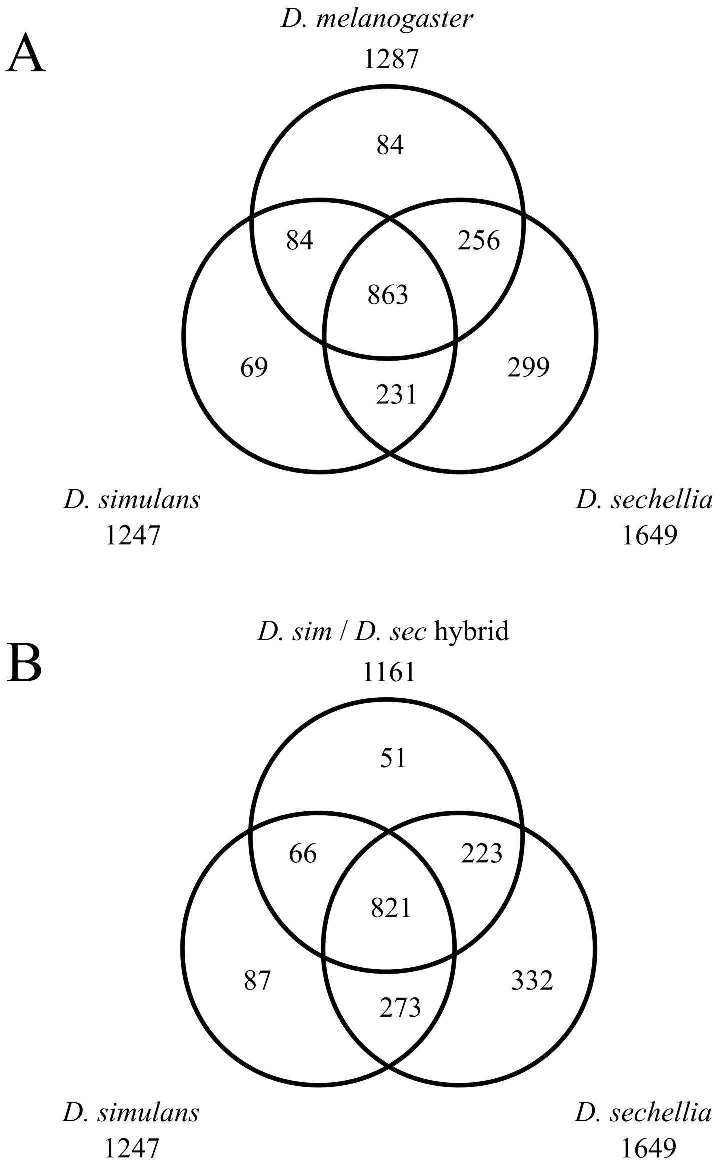

Supplement: Additional file 3 — Supplementary figure 1. Venn diagrams indicating the number of genes varying significantly in expression level over the four sampled developmental stages that are shared among the three pure species and the two parental species and the hybrids. [file 1741-7007-8-26-S3.PDF]
