# Supplementary material for: Molecular evidence for increased regulatory conservation during metamorphosis, and against deleterious cascading effects of hybrid breakdown in Drosophila
Source: BMC Biol. 2010 Mar 31;8:26. doi: 10.1186/1741-7007-8-26 (PMC2907589; doi:10.1186/1741-7007-8-26)

Additional Data File 4. Supplementary figure 2 - Boxplots comparing the distribution of between microarray spot replicate variances for each stage (L, larval; EP, early pupal; LP, late pupal; A, adult) within the three pure species (*D mel*, *D. melanogaster*; *D. sec*, *D. sechellia*; *D. sim*, *D. simulans*) and the *D. simulans* (♀) × *D. sechellia* (♂) F1 hybrids (Hybrid). Note that the mean variance is lower in *D. sechellia* during the L, EP, and LP stages as compared to all other species/hybrids (permuted Kruskal-Wallis rank sum test, $P < 2.2 \times 10^{-16}$). Also, no stages in the hybrids showed the highest mean variance in comparisons with the three pure species, despite the hybrid replicate spots representing biological replicates.

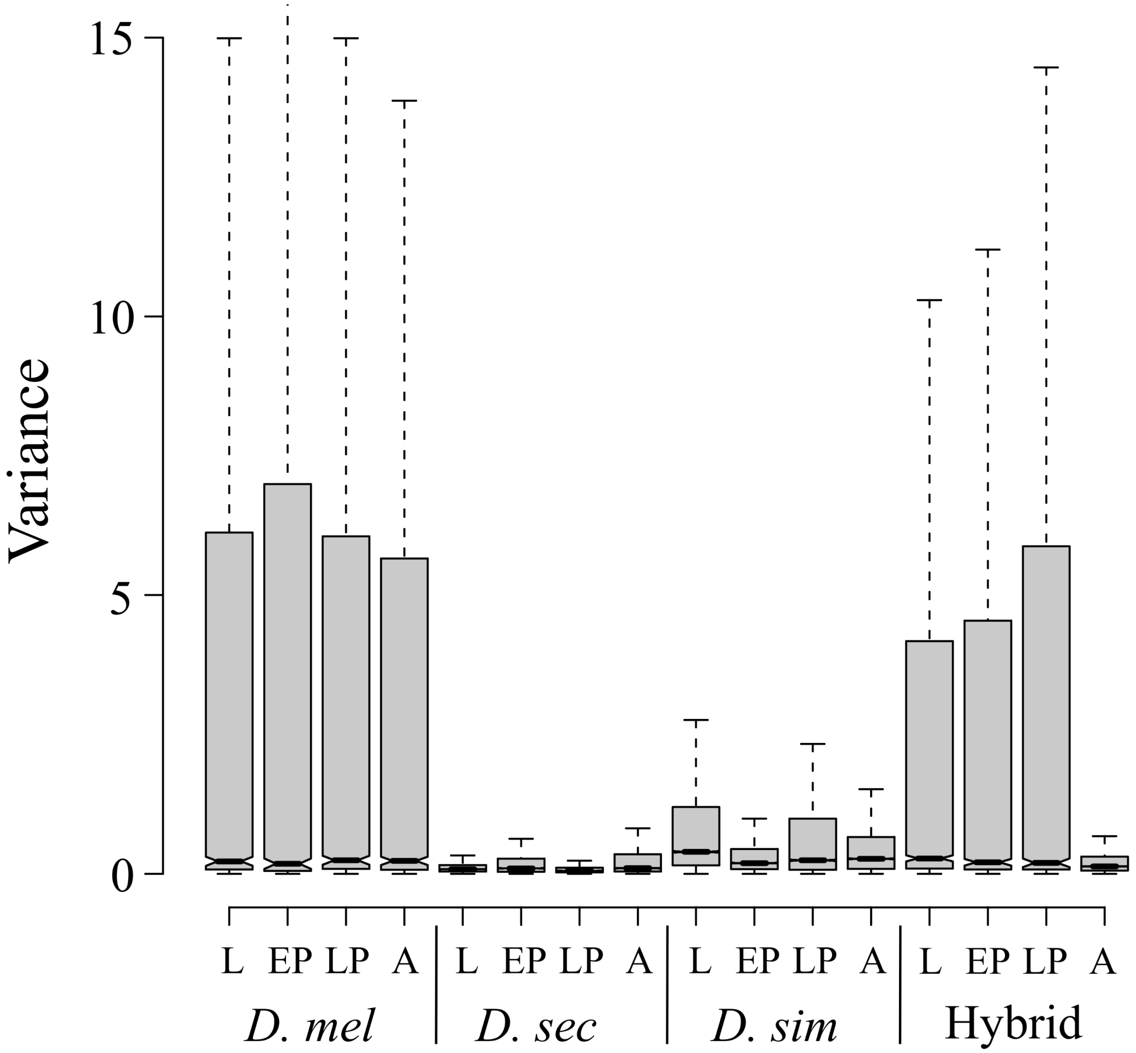

Supplement: Additional file 4 — Supplementary Figure 2. Boxplots comparing the distribution of between microarray spot replicate variances for each stage within the three pure species and the Drosphila simulans (male) × D. sechellia (female) F1 hybrids. [file 1741-7007-8-26-S4.PDF]
