# Supplementary material for: Molecular evidence for increased regulatory conservation during metamorphosis, and against deleterious cascading effects of hybrid breakdown in Drosophila
Source: BMC Biol. 2010 Mar 31;8:26. doi: 10.1186/1741-7007-8-26 (PMC2907589; doi:10.1186/1741-7007-8-26)

Additional data file 5. Supplementary figure 3 - Number of genes significantly differentially expressed in pairwise comparisons between *D. simulans*, *D. sechellia*, and the *D. simulans* (♀) × *D. sechellia* (♂) F1 hybrids (*D.sim*♀×*D.sec*♂) at each of the four sampled stages (out of 2,052 genes). The comparison between each species is divided into two segments, each indicating the number of genes that are expressed at a higher level in the species of the corresponding shade: grey, *D. simulans*; white, *D. sechellia;* and black, *D.sim*♀×*D.sec*♂ (see the legend in the upper-right hand corner of the figure). In the comparison between *D. simulans* and *D. sechellia* there are fewer genes differentially expressed during the larval stage than any other stage as indicated by the three asterisks (***) ($P < 0.001$). Similarly, in the comparison between *D. sechellia* and *D.sim*♀×*D.sec*♂ both the early and late pupal stages show significantly fewer genes that are differentially expressed as compared to the larval or adult stages again as indicated by the three asterisks ($P < 0.001$). All other between stage comparisons within the pairwise comparisons were not statistically significant. The daggers (†) indicate stages where the number of genes differentially expressed in the pairwise comparison between species is significantly lower than both other between species comparisons at the same stage ($P < 0.001$). Hash symbols (#) indicate comparisons where there is significant (χ^2 goodness of fit test, $P < 0.05$) asymmetry in the number of genes that are expressed at a higher level in one species vs. the other.

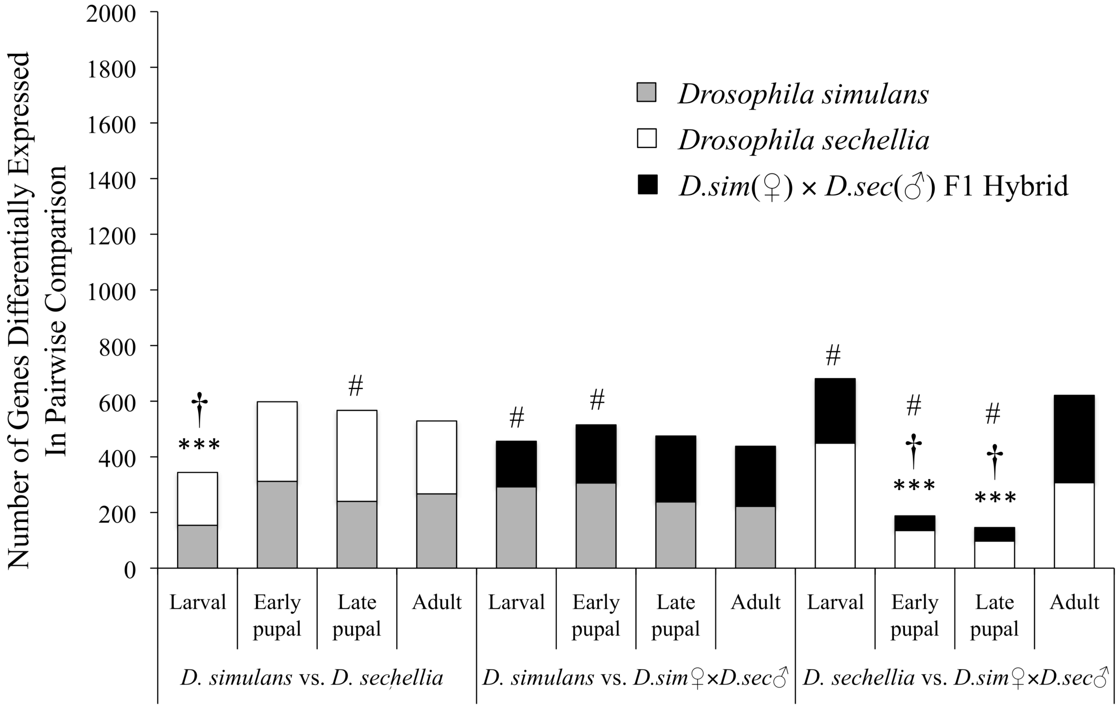

Supplement: Additional file 5 — Supplementary Figure 3. Number of genes significantly differentially expressed in pairwise comparisons between Drosphila simulans, D. sechellia, and the D. simulans (male) × D. sechellia (female) F1 hybrids (D. sim male × D. sec femalw) at each of the four sampled stages (out of 2052 genes). [file 1741-7007-8-26-S5.PDF]
