# Supplementary material for: Molecular evidence for increased regulatory conservation during metamorphosis, and against deleterious cascading effects of hybrid breakdown in Drosophila
Source: BMC Biol. 2010 Mar 31;8:26. doi: 10.1186/1741-7007-8-26 (PMC2907589; doi:10.1186/1741-7007-8-26)

Additional data file 6. Supplementary figure 4 - Scatter plots comparing the fold change in expression level between the hybrids and the two parental species for each of the three consecutive developmental transitions: (**A**) L to EP, (**B**) EP to LP, and (**C**) LP to A. The comparison and linear regression between *D. sechellia* and the hybrid is shown in red (circles), while the comparison and linear regression between *D. simulans* and the hybrid is shown in blue (circles). The slopes of the regression lines are all significantly different as determined by ANCOVA. The fold change in expression level between transitions in the hybrid is more significantly correlated to the *D. simulans* parent during the L to EP and LP to A transitions, while in the case of the EP to LP transition, the hybrid is more significantly correlated with *D. sechellia*.

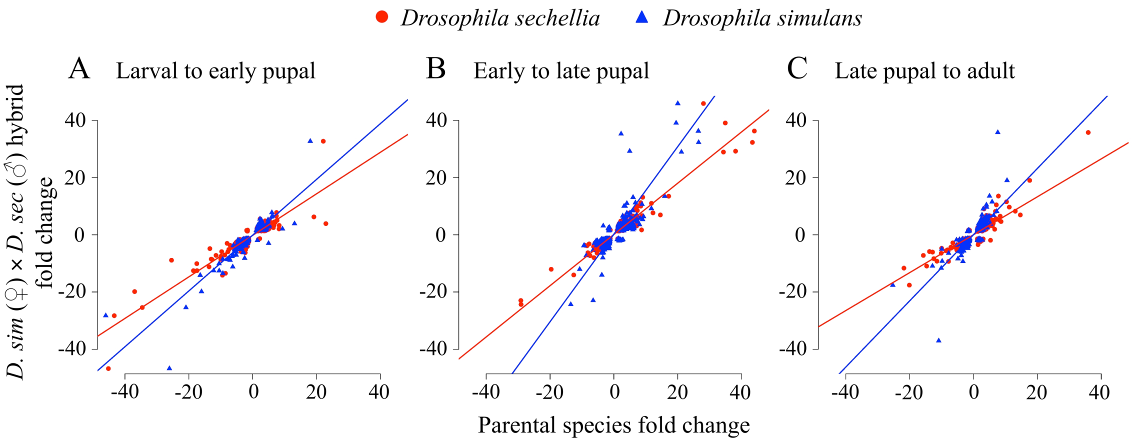

Supplement: Additional file 6 — Supplementary Figure 4. Scatter plots comparing the fold change in expression level between the hybrids and the two parental species for each of the three consecutive developmental transitions. [file 1741-7007-8-26-S6.PDF]
